# A dimensional measure of schizotypy: cross-cultural adaptation and validation of the Oxford-Liverpool Inventory of Feelings and Experiences short version for Brazilian Portuguese (O-LIFE-S)

**DOI:** 10.1590/2237-6089-2019-0013

**Published:** 2020-09-22

**Authors:** Letícia Oliveira Alminhana, Marcela Alves Sanseverino, Miguel Farias, Otávio Vendramin dos Santos, Wagner De Lara Machado, Gordon Claridge

**Affiliations:** 1 Laboratório de Estudos Avançados Multidisciplinares Universidade do Estado do Rio Grande do Sul Porto AlegreRS Brazil Laboratório de Estudos Avançados Multidisciplinares (LEAM), Universidade do Estado do Rio Grande do Sul (UERGS), Porto Alegre, RS, Brazil.; 2 Programa de Pós-Graduação em Psicologia Pontifícia Universidade Católica do Rio Grande do Sul Porto AlegreRS Brazil Programa de Pós-Graduação em Psicologia, Pontifícia Universidade Católica do Rio Grande do Sul (PUCRS), Porto Alegre, RS, Brazil.; 3 Department of Psychology and Behavioural Sciences Coventry University Coventry United Kingdom Department of Psychology and Behavioural Sciences, Coventry University, Coventry, United Kingdom.; 4 Department of Experimental Psychology University of Oxford Oxford United Kingdom Department of Experimental Psychology, University of Oxford, Oxford, United Kingdom.

**Keywords:** Schizotypy, adaptation, O-LIFE

## Abstract

**Introduction:**

The Oxford-Liverpool Inventory of Feelings and Experiences (O-LIFE) is a widely-used scale, and the first to include a dimensional approach to understanding schizotypy.

**Objective:**

To adapt the short version of the O-LIFE (O-LIFE-S) into Brazilian Portuguese.

**Method:**

a) Two independent bilingual professionals translated the original instrument into Brazilian Portuguese; b) a third bilingual professional summarized the two translations; c) a fourth bilingual expert translated the Portuguese version back into English; d) this back-translation was adjusted by a committee of psychology experts; e) a pilot study was conducted with 10 participants from the general population.

**Results:**

O-LIFE-S was considered ready to be used in a formal validation study in Brazil.

**Conclusion:**

The scale appears to cover the dimensional approach to schizotypy. However, a future validation study needs to be conducted to determine the internal consistency and reliability of the Brazilian Portuguese version of the O-LIFE-S .

## Introduction

Bleuler (1857-1939) was the first author to identify “moderate schizophrenia” as a discrete expression of psychosis. Since then, there have been several interpretations of schizophrenia, one of which is a dimensional model of psychosis encompassing healthy expressions of psychotic-like features, particularly as a personality trait – schizotypy.^1 , 2^

Claridge and colleagues describe schizotypy as a personality trait that, within a general population, is underpinned by creativity, spirituality, and divergent thinking, but in its extreme form is a personality disorder.^3^ Schizotypy is thus a multi-factorial construct, which can manifest as well-being or mental illness, depending on environmental context and life events.^4^

Following this characterization, one of the most widely-used schizotypy scales, the Oxford-Liverpool Inventory of Feelings and Experiences (O-LIFE)^4 , 5^ comprises four dimensions:

1. Unusual experiences: magical ideation, altered perceptions and sensations, hypersensitivity to smells and sounds, auditory hallucinations, and pseudohallucinations.
2. Cognitive disorganization: problems with attention, concentration, decision making, lack of purpose, and social anxiety.^5 , 6^
3. Introvertive anhedonia: lack of pleasure in physical or social contact, avoidance of intimacy, schizoid solitude, and flat affect.^6^
4. Impulsive nonconformity: disinhibition, impulsivity, violence, and recklessness.^5^

### The need for a schizotypy measure with a dimensional approach

Although the Diagnostic and Statistical Manual of Mental Disorders, 5th edition (DSM-5) has maintained the categorical/symptom-based approach, it has also included many transnosological specifiers, symptom or syndrome-related severity, and dimensional assessments.^7^ Schizotypal personality disorder is included in the DSM-5 chapter on schizophrenia spectrum disorders.^8^ In addition, the manual also presents two different approaches to personality disorders: a) categorical (similar to the Diagnostic and Statistical Manual of Mental Disorders, 4th edition, Text Revision [DSM-IV-TR]); and b) categorical-dimensional (hybrid), known as the alternative model for personality disorders.^9^ The aim of this alternative model was to preserve clinical practice, but also to remedy some of the major problems of categorical diagnosis of such disorders.^9^

Several studies using this dimensional approach to schizotypy have robustly showed that psychotic-like experiences can be associated with mental health and well-being outcomes (benign schizotypy).^10 , 11^ This more sensitive and nuanced dimensional measure of schizotypy offers a more comprehensive understanding of the construct, in its healthy and pathological components.

The only instruments for assessment of schizotypy that has been adapted for the Brazilian context is the Schizotypal Personality Questionnaire (SPQ).^12 , 13^ The SPQ was developed to assess schizotypal personality disorder based on the diagnostic criteria from the Diagnostic and Statistical Manual of Mental Disorders, 3rd edition, Revised (DSM-III-R),^13^ which entails a taxonomic and non-dimensional approach to schizotypy. The SPQ considers 3 factors – cognitive-perceptual deficits, interpersonal deficits, and disorganization^12 - 14^ – which overlap with the first three factors of the O-LIFE. In addition to including a 4th dimension, referring to “non-social behavior,” the O-LIFE also offers the benefit of a dimensional theoretical framework. Given the cultural prevalence of unusual experiences in Brazilian culture, the O-LIFE is the ideal instrument to assess schizotypal traits in the general population.^10^

### The value of O-LIFE in the literature or state-of-the-art, including application of O-LIFE

A number of researchers around the world have been translating, adapting and validating O-LIFE, either the short version (O-LIFE-S) or the full version. To the best of our knowledge, O-LIFE has already been translated into: Spanish,^15^ German,^16^ French,^17^ Polish,^18^ and Hungarian.^19^ With the exception of the Spanish version, all of these studies used the full version of O-LIFE. Recently, the O-LIFE has been adapted for American English in a study whose authors also made changes such as substituting the dichotomous scale with a 5-point Likert scale and used it to develop a parent-report scale for assessment of children.^20 , 21^

As a result, the O-LIFE has become an important tool for correlating schizotypal traits with other approaches to psychiatric disorder assessment. For instance, a group of Spanish researchers correlated O-LIFE scores with handedness (measured by Annet’s Hand Preference Questionnaire), in order to demonstrate that mixed and ambiguous handedness might be related to proneness to psychosis in an adolescent sample.^22^ The authors supported the hypothesis that schizophrenia is a neurodevelopment disorder that originates when brain asymmetries are being established. Likewise, Grant et al. reported significant genetic associations (regarding dopamine-related candidate polymorphisms of schizotypy) with O-LIFE subscales scores, contributing insights to the newest dopamine hypothesis.^16^

In contrast, studies have also been using O-LIFE as a psychometric instrument to associate schizotypal traits with health parameters. One good example would be a paper published by Farias et al.,^23^ in which they explore correlations between O-LIFE scores and mental health indicators in spiritual believers divided into two groups, traditional and modern spirituality.^11^ Furthermore, the authors’ overall conclusion was that there are strong correlations between modern spirituality (or benign schizotypy) and good mental health and general well-being.

Similarly, Tabak & Weisman de Mamani^21^ explored latent profiles that emerged in a nonclinical sample and compared psychological well-being across profiles.^21^ Surprisingly, they found similar characteristics to the modern spirituality group in one of the six profiles that emerged.^11^ This profile had a high score only in the unusual experience subscale of the O-LIFE, but still exhibited similar scores to the profiles of people without any high scores and those with average scores across all subscales in the domains of autonomy, quality of life, positive relations with others, personal growth, purpose in life, and self-acceptance.^21^

Thus, the O-LIFE is proving to be a valid and reliable psychometric tool used worldwide and allowing researchers not only to evaluate schizotypy as a whole, but also to assess its traits through the subscale scores. Taking into account each individual subscale, it is important to note, however, that impulsive nonconformity usually scores lowest in internal consistency analysis coefficients.^15 , 16 , 18 , 19^

### Objective and hypothesis

By all accounts, both the full and the short versions of the O-LIFE have a well-established currency in recent literature. The short scale (O-LIFE-S) was successfully developed in order to retain, and even maximize, the degree of genotypic variance captured by items, without compromising scale content.^6^ To the best of our knowledge, there are no scales in Portuguese, particularly for research use in Brazil, that are capable of measuring schizotypy traits in clinical and, most importantly, nonclinical samples. Therefore, the main purpose of this study is to adapt and validate the O-LIFE for the Brazilian setting.

## Method

### Instrument

Short version of the O-LIFE (O-LIFE-S)

The O-LIFE is the most comprehensive test of schizotypal traits available in the English language.^4^ Factor analysis of the first English version included use of 15 other personality inventories.^4 , 5^ The O-LIFE-S comprises 43 items, divided into 4 subscales:

1. Unusual experiences (UnEx) – 12 items, e.g., “Have you ever thought that you had special, almost magical powers?”
2. Cognitive disorganization (CogDis) – 11 items, e.g., “Are you easily distracted from work by daydreams?”).
3. Introvertive anhedonia (IntAn) – 10 items, e.g., “Have you often felt uncomfortable when your friends touch you?”).
4. Impulsive nonconformity (ImNon) – 10 items, e.g. “Do you stop to think things over before doing anything?”). The first publications describing the original scale in English demonstrated that it had high internal consistency (0.72-0.89) and reliability for each of its 4 subscales.^5 , 6^ The O-LIFE-S also showed good reliability and content and concurrent validity.^6^

### Procedures

Although there is no absolute consensus on how to adapt and validate scales for different languages and cultural contexts, we followed the guidelines offered by various experts.24 Accordingly, the procedures involved in adaptation of the O-LIFE-S were as follows:

- The original instrument was translated by two independent bilingual professionals.
- A third bilingual professional summarized the two translations. This synthesis was analyzed by a committee of psychology experts, taking into account the theoretical framework and the Brazilian cultural context.
- A fourth bilingual expert translated the Portuguese version back into English.
- This back-translation was revised by the committee of psychology experts and sent to Gordon Claridge (who is one of the authors of the original scale), to ensure the reliability of the content and meaning of the translated and adapted version.
- A pilot study was conducted with the general population.
- Final adjustments were made after feedback from participants.
- The instrument was considered ready to be used in a formal validation study in Brazil.

### Ethics approval

This study was approved by the ethics committee at the Pontifícia Universidade Católica do Rio Grande do Sul (PUCRS) under registration number2.834.926. In the pilot study, the O-LIFE-S was administered to 629 volunteers who did not receive any remuneration or additional credits for participation in the study. Inclusion/exclusion criteria for participants were as follows: over the age of 18 years and at minimum of 12 years in formal education (formal education is needed to understand the questions in order to answer the instrument). A link to the Qualtrics platform was sent via e-mail to the university’s students and published on social media with a short message explaining the purpose of the study and the inclusion criteria for answering the questionnaire online. Volunteers who decided to participate would follow the link and, before answering the O-LIFE, they were asked to read and accept a consent agreement.

### Analysis

Confirmatory factor analysis^25^ was conducted to investigate the relationship between the hypothetical dimensional model of O-LIFE items and the data collected in the present study. A four-factor, oblique model, with no item covariation was developed to represent the underlying structure of the O-Life items and latent traits. Diagonally weighted least squares (DWLS) with the robust standard errors estimation method was used to verify the fit of the model to the data. This estimation method is preferred when variables are not continuous but have an ordered or dichotomous structure.^26^ We considered the following fit indexes to assess model quality: comparative fit index (CFI ≥ 0.90), Tucker-Lewis index (TLI ≥ 0.90), and root mean square error of approximation (RMSEA ≤ 0.06). We also examined both the items’ factor loadings (each item’s relation with the latent factors) and their thresholds (a proxy for the likelihood of an item being endorsed) to interpret their roles in the latent characteristics being measured. Items which did not meet either the factor loading ≥ |0.30| or p ≤ 0.05 criteria were removed, and a new re-specified model was estimated. We used the composite reliability^27^ formula (square of the sum of the standardized factor loadings divided by the same term plus measurement error) to assess the internal consistency for each of the subscales.

## Results

This version resulted from a first empirical investigation with a sample of 629 adults (66.6% female; mean age 36.04, standard deviation = 13.4). Confirmatory factor analysis indicated that items 5, 28, 30, 31, and 42, did not meet minimum psychometric criteria such as standardized factor loadings higher than 0.30. Table 1 shows the final version of the O-LIFE-S, with 38 items instead of the 43 on the original scale, in Brazilian Portuguese.

**Table 1:** Factors found in the confirmatory analyses and their items

Factor	Item number
Unusual experiences ( *experiências incomuns* )	1, 5, 9, 14, 17, 22, 26, 27, 28, 29, 34, 36
Cognitive disorganization ( *desorganização cognitiva* )	2, 8, 11, 15, 16, 21, 25, 32, 35, 37, 38
Introvertive anhedonia ( *anedonia introvertida* )	3, 7, 13*, 18*, 20, 24, 30*
Impulsive nonconformity ( *não-conformidade impulsiva* )	4, 6, 10, 12, 19, 23*, 31, 33

Dimensions in English and Brazilian Portuguese and items for each dimension will be described afterwards.

Scores: 1 for yes and 0 for no, except for items marked with*, which have inverted scores: 0 for yes and 1 for no.

Table 2 shows the characteristics of the sample. Most of the participants (48.65%) were from the South of Brazil, where our university is located. Pearson correlation coefficients between the subscales and age showed moderate negative associations with all subscales, but cognitive disorganization (r = -0.276) and impulsive nonconformity (r = -0.288) had the largest coefficients. Educational level also had negative associations with all subscales, strongest with cognitive disorganization (r = -0.240). All associations were significant to p < 0.001.

**Table 2:** Sample characteristics

Characteristics	n	%
Total sample	629	
Sex (female/male)	419/147	66.61/23.37
Age, years (mean, SD)	36.09 (13.45)	-
Marital status		
Single	285	45.31
Married	234	37.20
Divorced	41	6.52
Educational level		
Middle school	36	5.72
Undergraduate	182	28.94
Graduate	132	20.99
Post-graduate	206	32.75
Occupation		
Student	169	26.87
Unemployed	23	3.66
Employed	181	28.78
Self employed	147	23.37
Retired	35	5.56
Brazilian region of origin		
South	306	48.65
Southeast	70	11.13
Northeast	25	3.97
Religion		
No religion	154	24.48
Non-practicing Catholic	103	16.38
Practicing Catholic	52	8.27
Spiritist	132	20.99
Umbandist	20	3.18
Other religion	77	12.24

n = number of subjects; SD = standard deviation.

Table 3 shows mean subscale scores compared by sex (male/female). Unusual experiences scores were higher in women.

**Table 3:** Subscale scores compared by sex

	All	Female	Male	p-value
UnEx	4.85 ± 3.00	5.04 ± 2.97	4.34 ± 3.05	0.019
CogDis	4.37 ± 3.01	4.47 ± 2.98	4.18 ± 3.14	0.338
IntAn	2.06 ± 1.21	2.09 ± 1.26	1.99 ± 1.03	0.358
ImpNon	2.27 ± 1.76	2.28 ± 1.76	2.25 ± 1.77	0.871
Total	13.32 ± 6.58	13.70 ± 6.47	12.49 ± 6.78	0.061

CogDis = Cognitive Disorganization; ImpNon = Impulsive Nonconformity; IntAn: Introvertive Anhedonia; UnEx = Unusual Experiences.

The values shown in the table are mean ± standard deviation.

The only p < 0.05 is presented in bold font.

**Figure 1:**
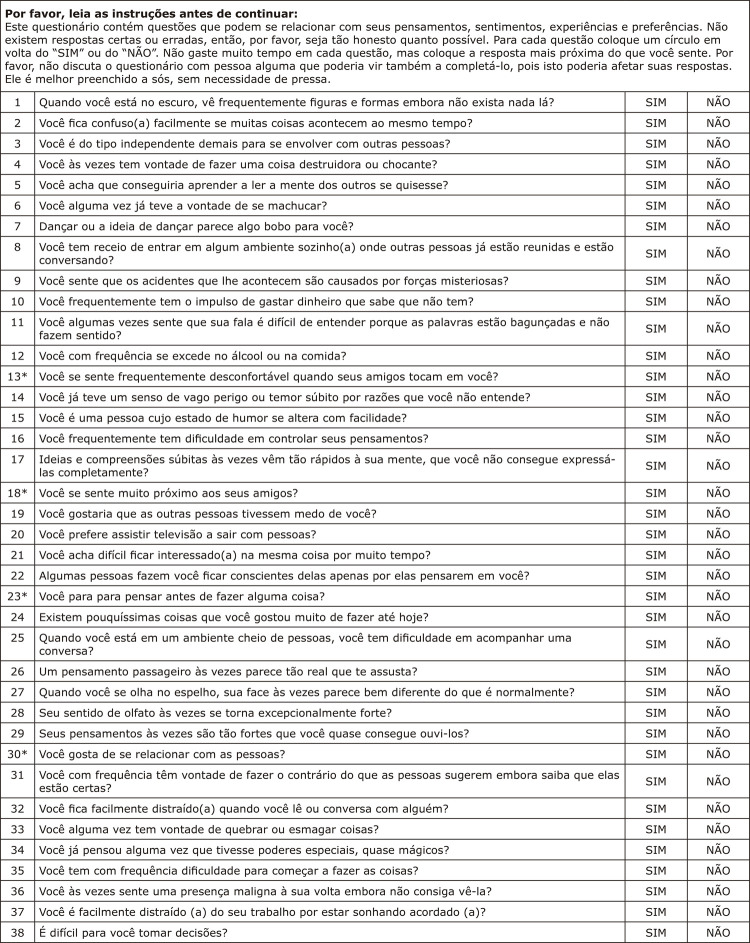
Final version of the O-LIFE-S (38 items) in Brazilian Portuguese. Title in Brazilian Portuguese: Inventário Oxford-Liverpool de Sentimentos e Experiências – O-LIFE. In Portuguese, “sim” means yes and “não” means no, since these are yes or no questions. Scores: 1 for yes and 0 for no, except for items marked with*, which have inverted scores: 0 for yes and 1 for no.

Figure 1 shows the final version of O-LIFE-S in Brazilian Portuguese, entitled: Inventário Oxford-Liverpool de Sentimentos e Experiências – O-LIFE. The confirmatory factor model comprising 43 items in four factors did not achieve adequate fit indices (χ^2^ (854) = 1,286.4; CFI = 0.88, TLI = 0.88, RMSEA = 0.031). The model had low variance explained (CFI and TLI) despite low residuals (RMSEA). Items 5, 28, 30, 31, and 42 on the original scale had factor loadings below the cut-off criterion of 0.3 and not statistically different from zero. These five items were removed and a new, respecified model was evaluated. The adjusted model produced a better fit to the data (χ^2^ (659) = 1,052.9; CFI = 0.92, TLI = 0.91, RMSEA = 0.034). After removal of the five items from the original inventory, the remaining items were renumbered from 1 to 38.

**Figure 2:**
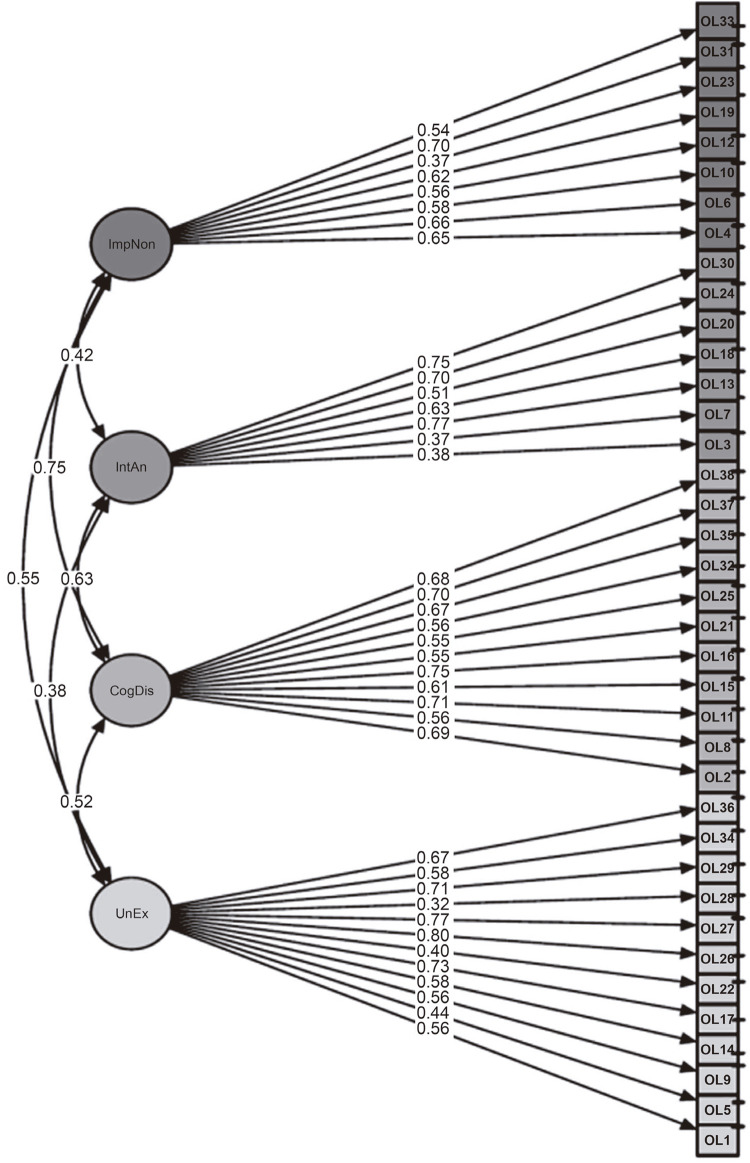
Standardized factor loadings and factor correlations of the O-LIFE-S in a Brazilian sample. CogDis = cognitive disorganization; ImpNon = impulsive nonconformity, IntAn = introvertive anhedonia; OL = Oxford-Liverpool Inventory of Feelings and Experiences (O-LIFE); UnEx = unusual experiences. The arrows between factors represent the correlation between each factor and the arrows between the factors and each item represents the factor loadings. The boxes on the right represent each item, where OL means OLIFE and the number indicates the number of the item. The horizontal line on the right side of each item’s box represents its threshold, in which the higher the line the less likely it is that the item will be endorsed.

Figure 2 shows the standardized factor loadings and factor correlations. The diagram also illustrates the threshold parameter on the vertical axis of each item’s box. The higher the position of the line on vertical axis in Figure 2 , the more severe the item (i.e., the participant must have high levels of the trait to endorse the question as describing his/her behavior). Composite reliability, calculated with the formula: (sum standardized factor loadings)^2^ / (sum standardized factor loadings)^2^ + measurement error, was adequate (composite reliability ≥ 0.7) for all subscales (UnEx = 0.88, CogDis = 0.88, IntAn = 0.80 and ImpNon = 0.81).

## Discussion

This study aimed to adapt and validate the O-LIFE-S for the Brazilian setting. The process of adapting a schizotypy measure into Brazilian Portuguese started with a consideration of the theoretical framework of psychoticism as a personality trait continuum, associated with well-being as a benign form of schizotypy or with mental illness (schizotypal personality disorder) at the extreme of this continuum. Other measures of schizotypy do not allow for a dimensional assessment because they are attached to categorical Diagnostic and Statistical Manual of Mental Disorders, 4th edition (DSM-IV), criteria for the schizotypy personality disorder.

The translation and semantic adaptation of the O-LIFE into Brazilian Portuguese was considered appropriate for our cultural context. The dimensional and personality trait approach to schizotypy and its four sub factors seems to be covered by the 38 items of the O-LIFE-S. We chose to maintain the four-factor solution (UnEx, CogDis, IntAn, and ImNon).^4 , 6 , 18^ First, because it was a theoretical path where the psychotic continuum covers positive and negative symptoms, disorganization, and impulsivity/aggression.^6^ Second, because there is an empirical consensus favoring a three or four-factor solution over one or two-factor models.^15^

Participants of the study were, mostly, women, of middle age, single, graduates, employed, and from the south of Brazil. Unusual experiences was the only subscale in which women scored statistically higher than men. These results are in line with a large study comparing the phenotypic expression of schizotypal traits in 12 countries (Greece, United Kingdom, United States, China, Australia, Spain, Switzerland, New Zealand, Canada, Italy, Norway, Belgium, and Tunisia).^28^ As in our sample, most of the participants in these 12 countries were women and they scored higher in the positive dimension (UnEx). We observed a significant decrease by age in all subscales of schizotypy. Fonseca-Pedrero et al.^28^ observed the same decrease, but only associated with the positive traits. Similarly, previous Brazilian studies also reported a majority of women, of middle age, and with an educational level between undergraduate and graduate.^29 , 30^ The subscale with the highest score in those Brazilian studies was UnEx, followed by CogDis, as we found in our sample.

In the confirmatory factor analysis, five specific items from the original inventory had factor loadings below 0.3. Additionally, we realized that all of these items were not very clear and could be tricky to understand for Brazilians. We decided to reduce the 43-item short form to a 38-item solution and the adjusted model produced a better fit to the data, confirming analyses and empirical observations. As far as we know, no other cross-cultural adaptation has resulted in exclusion of the same O-LIFE-S items. Composite reliability was adequate for all subscales, providing original information about schizotypy using a fully dimensional approach with a nonclinical sample, as we can see elsewhere.^15^

Figure 2 illustrates standardized factor loadings in conjunction with the correlations between subscales and item thresholds (severity level of each item). The strongest association between factors was between ImNon and CogDis (r = 0.75). According to Mason et al.,^5^ the ImNon dimension is related to disinhibition, impulsivity, and violence, while CogDis is linked to impairments in attention and decision making and lack of purpose and social anxiety. We hypothesize that impulsivity and attention problems are intimately related to social problems and anxiety. Along these lines, a recent study showed that patients with social anxiety disorder and problems with impulse control had higher severity symptoms of attention-deficit/hyperactivity disorder.^31^

On the other hand, the lowest subscale correlation was between IntAn and UnEx (r = 0.38). IntAn is related to a lack of pleasure in physical or social contact and flat affect, while UnEx is associated with magical ideation and auditory and pseudo-hallucinations.^5^ These are dimensions expressing negative and positive symptoms, respectively. There are many studies showing the difference between negative and positive schizotypy.^32 - 34^ Interestingly, some studies have showed a correlation between positive schizotypy and health and\or well-being, possibly indicating a trait of benign schizotypy.^10 , 11 , 35^

Of note in Figure 2 is the difference between factor loadings and threshold parameters in each item’s box. Item 26 is a good example (“Does a passing thought ever seem so real it frightens you?” – translated into Brazilian Portuguese as “Um pensamento passageiro às vezes parece tão real que te assusta?”). This question had the highest factor loading in the confirmatory factor analysis, onto the UnEx subscale (r = 0.80). This means that question 26 is very sensitive to any change in this subscale. In other words, a small change in the positive schizotypy trait will have an impact on the way a person answers question 26. However, the threshold (represented by the horizontal line drawn on the vertical line on the right-hand side of the box for item 26, in Figure 2 ) is not very high, which indicates that this is not a very severe item, meaning it is not so unlikely that a person will endorse this question.

Another example is item 30, which loaded strongly onto the subscale Introverted Anhedonia (r = 0.75) and has the highest threshold in the analysis ( Figure 2 ). Question 30 is: “Do you like mixing with people?” (translated into Brazilian Portuguese as “Você gosta de se relacionar com as pessoas?” It is reverse coded, meaning that someone with high introvertive anhedonia will answer “no” and will score 1 for the item). For instance, we can see that not only can question 30 be impacted by some change in the negative dimension of schizotypy (IntAn), but also that it is very unlikely that people will endorse score 1 on this item, so it is indicative of the severity of the trait. To score 1 on question 30 would mean that the subject demonstrates a high absence of social bonding and a lack of affect. Cultural aspects could also play an important role in the threshold of this item, although we couldn’t find any literature exploring these characteristics. Nonetheless, Brazil is well-known for its sociable and friendly people.

Threshold parameters are very useful for understanding the continuum of psychotic traits and schizotypy in a dimensional model. The O-LIFE-S synthesizes the spectrum of characteristics from lower to higher severity and risk for psychosis. For instance, traits with low threshold, such as a “Does a passing thought ever seem so real it frightens you?” (question 26) are aligned with one end of the spectrum, underpinned by healthy characteristics such as creativity, spirituality and divergent thinking.^3^ At the opposite extreme of the same spectrum of schizotypy, we see a trait with a high threshold, associated with an absence of pleasure in social relationships (anhedonia – question 30).

The dimensional perspective of schizotypy cannot be found in scales such as the Schizotypal Personality Questionnaire (SPQ),^12 , 13^ linked to categorical models and the DSM III-R checklist for personality disorder. Contemporary approaches to proneness to psychosis assume schizotypy is a personality trait that is a complex, dynamic, and multi-factorial construct.^8 - 10^ The O-LIFE does not assume a pathological perspective a priori, as we can see from the instrument’s name. Environmental interactions will lead feelings and experiences, as personality traits, to well-being or to mental illness, according to a broad spectrum of possibilities.^4^

This study includes certain limitations that should be considered. First, our sample is not representative of all Brazilian citizens, but we were able to assess a sample from the South and the Southeast of our large country, which itself is of great value. Nevertheless, we recommend researchers from the North and Northeast of Brazil make adjustments as they see fit.

## Conclusions

In summary, the present study was able to validate a dimensional tool to assess schizotypy for the Brazilian setting. The O-LIFE-S achieved an optimal correspondence with our language and is well-adapted to our culture. Fortunately, this allows Brazilian researchers and clinicians to investigate the schizotypy continuum in Brazil. Future studies should compare clinical and nonclinical populations cross-culturally.
